# 5-Aminovaleric acid betaine predicts impaired glucose metabolism and diabetes

**DOI:** 10.1038/s41387-023-00245-3

**Published:** 2023-09-20

**Authors:** Linus Haberbosch, Sylwia Kierszniowska, Lothar Willmitzer, Knut Mai, Joachim Spranger, Lukas Maurer

**Affiliations:** 1 Department of Endocrinology and Metabolism, Charité – Universitätsmedizin Berlin, 10117 Berlin, Germany; 2 Berlin Institute of Health at Charité – Universitätsmedizin Berlin, BIH Biomedical Innovation Academy, BIH Charité Junior Digital Clinician Scientist Program, Charitéplatz 1, 10117 Berlin, Germany; 3 metaSysX GmbH, Am Mühlenberg 11, 14476 Potsdam-Golm, Germany; 4 Charité – Universitätsmedizin Berlin, Charité Center for Cardiovascular Research, 10117 Berlin, Germany; 5 DZHK (German Centre for Cardiovascular Research), partner site Berlin, Berlin, Germany

**Keywords:** Type 2 diabetes, Metabolic syndrome

## Abstract

**Background:**

5-Aminovaleric acid betaine (5-AVAB) has recently been identified as a diet and microbial-dependent factor inducing obesity and hepatic steatosis in mice fed a Western diet. Accumulating evidence suggests a role in metabolic dysfunction associated with obesity, diabetes, and fatty liver disease. However, whether 5-AVAB plays a role in human disease is unclear, and human data are sparse.

**Methods:**

We measured circulating 5-AVAB serum levels in 143 individuals with overweight or obesity participating in a randomized intervention study (NCT00850629) investigating the long-term effect of a weight maintenance strategy after diet-induced weight reduction.

**Results:**

Higher 5-AVAB serum levels correlate with worse estimates of obesity, glucose metabolism, and hepatic steatosis after weight loss. Furthermore, higher 5-AVAB levels after weight loss independently predict detrimental changes in glucose metabolism 18 months after the successful weight reduction.

**Conclusion:**

Our human data supports previous findings in rodents indicating a relevant, potentially disadvantageous function of 5-AVAB in the context of metabolic dysbalance.

## Introduction

The metabolic syndrome, typically characterized by impaired glucose metabolism, abdominal obesity, hypertension, and dyslipidemia, accounts for the largest burden of non-communicable diseases worldwide [1]. It can lead to the development of type 2 diabetes (T2DM), non-alcoholic fatty liver disease (NAFLD), and cardiovascular disease [2], among others. Therefore, identifying and preventing the causes and consequences of metabolic syndrome is a major challenge facing global healthcare [3].

Weight loss plays an important role in preventing and treating metabolic syndrome. However, while short-term weight reduction can be achieved easily, long-term weight loss is much more difficult to attain. As this long-term weight loss is critical for successful prevention and treatment, predictive markers are necessary.

Trimethylated compound 5-Aminovaleric acid betaine (5-AVAB, HMDB0240732), also called δ-valerobetaine, has recently gained importance as a possible metabolic marker [4]. While 5-AVAB is a microbial metabolite, it is also present in a variety of food sources including ruminant meat and milk [5, 6], and can be influenced by a whole-grain diet [7]. Recently Liu et al. provided mechanistic evidence for the role of 5-AVAB as a dietary obesogen [8]. Their data shows that 5-AVAB inhibits mitochondrial fatty acid oxidation by decreasing cellular mitochondrial long-chain acyl-coenzyme A [8]. In mice fed a western diet, 5-AVAB led to greater weight gain including an increase in visceral adipose tissue (VAT), and hepatic steatosis [8]. In humans, increased plasma 5-AVAB was associated with increased VAT, BMI, and incidence of hepatic steatosis [8]. These data suggest a role for 5-AVAB in the etiology of metabolic syndrome as well as the development of obesity and NAFLD [4]. In light of these findings, we decided to analyze 5-AVAB measurements in a weight loss-weight maintenance study and investigate it as a predictive factor for improvement in glycemic control. The study’s initial objective was to identify predictors of weight regain and its associated comorbidities after a successful short-term weight loss intervention. Following this concept, we investigated to what extent 5-AVAB levels before and after weight loss predicted future glycemic control.

## Materials and methods

### Participants

We measured 5-AVAB levels in a randomized controlled trial (Maintain-Adults) performed between 2010 and 2016. Initially, a total of 223 subjects with overweight or obesity were screened for participation. Subjects (156 total; 120 female and 36 male; BMI ≥ 27 kg/m^2^) were enrolled in the pretrial weight loss phase, and 143 participants who lost at least 8% of their body weight during the weight loss phase were randomized. Further information on recruitment and patient characteristics has been reported elsewhere [9].

### Study approval

The study protocols were approved by the Ethics Committee of the Charité—Universitätsmedizin Berlin (“Ethikkommission der Charité—Universitätsmedizin Berlin”), EA2/017/09) and all subjects gave written informed consent. The trial was registered at ClinicalTrials.gov (NCT00850629).

### Study design

Subjects were analyzed before (T-3) and after (T0) a standardized 12-week dietary weight reduction program. Afterward, subjects were randomized to a 12-month lifestyle intervention or a control group. Randomization was done via a stratified randomization list, with stratification regarding gender and body weight at T0 (three BMI strata). Study nurses and physicians but not participants were blinded to group assignment. After 12 months (T12) no further intervention was performed until 6 months later (T18). The lifestyle intervention comprised 8 weeks of caloric restriction (800 kcal/day) with total food replacement, followed by 4 weeks with an energy-reduced healthy diet. All participants achieved an initial weight reduction of >8% body weight and underwent subsequent randomization to a 12-month multimodal lifestyle intervention versus follow-up. A schematic of the study design is provided in Supplemental Fig. 1. Detailed information is provided in a previous publication [9].

### Procedures

We included data from the comprehensive phenotyping performed before (T-3) and after (T0) weight loss as well as at 18 months follow-up (T18). A detailed methodological overview was published previously [9]. In short, concomitant anthropometric and metabolic phenotyping at all time points included the following parameter sets:

- Obesity: BMI, total fat mass, lean body mass (bio-impedance measurement),
- Glucose homeostasis: HbA1c, insulin sensitivity (ISI clamp—hyperinsulinemic-euglycemic clamp (not at T18), HOMA-IR, oral glucose tolerance test [10]), insulin secretion (AUC Insulin/AUC Glucose [11], disposition index—oral glucose tolerance test [12]),
- Hepatic steatosis: fatty liver index (FLI), liver fat score (LFS) [13].

### Laboratory tests

Laboratory analyses were performed using established methods. Details including the inter- and intra-assay coefficients of variation are provided in Mai et al. (2018) [9]. Relative 5-AVAB levels were determined using a high-resolution mass spectrometric profiling system (metaSysX GmbH). The identification of 5-AVAB was confirmed with the use of the MS/MS fragment spectrum of the feature 160.1331. The fragmentation spectrum and extracted ion chromatogram are provided in Supplemental Fig. 2. A full methodological description is provided in Supplementary Text 1.

### Statistical analysis

Due to the lack of normal distribution of 5-AVAB values determined by the Shapiro–Wilk test, we log-transformed the data. Normal distribution and homoscedasticity of the data were then confirmed via the Shapiro–Wilk and Levene’s test, respectively.

Correlations at T-3 and T0 were calculated as partial correlations corrected for age and sex. The regression analyses were performed as listwise linear regressions with the independent variables log5-AVAB, age, sex, and randomization and the dependent variable of either T18 BMI, T18 HbA1c, or T18 LFS. These regression analyses were repeated with the added independent variable of BMI change (T18–T0) (for analysis of HbA1c and LFS) as well as either T0 BMI, T0 HbA1c, or T0 LFS, correspondingly.

For the following analyses, we defined the variable “diabetes status”, classified as 1 = “no diabetes”, 2 = “impaired fasting glucose or impaired glucose tolerance” or 3 = “type 2 diabetes mellitus”. The multinomial logistic regression analyses were performed with diabetes status change (−1 = remission, 0 = no change, 1 = progression) as the dependent variable and the independent variables log 5-AVAB (in tertiles), age, sex and randomization and BMI change (T18–T0). All analyses mentioned above were repeated after the exclusion of patients with antidiabetic medication.

For the effect of the weight loss intervention on logVB, we performed a two-sided *t*-test.

Statistical procedures were performed using SPSS version 22.0 (SPSS Inc., Chicago, IL), SAS software, version 9.4 (SAS Institute), and the R software package. Raw values were reported and plotted unless otherwise mentioned.

Details of all statistical analyses are described in the supplementary data.

## Results

### Cohort characteristics

The 143 participants were metabolically characterized directly and 18 months after a weight loss intervention [9, 14]. The complete protocols of this study are described in detail in a previous publication [9]. For our analysis, we selected all participants with biosamples available to determine 5-AVAB relative levels at T-3 and T0. The patients’ characteristics and metabolic profiles are presented in Table 1.

**Table 1:** Patient characteristics.

Characteristics	T-3		T0		T18	
	*N*	Values	*N*	Values	*N*	Values
Age (years)	143	50.5 ± 12.6	143	50.8 ± 12.6	112	53.9 ± 12.2
Sex, *N*	143					
Male	31	21.5%				
Female	112	77.8%				
BMI (kg/m^2^)	143	37.3 ± 6.1	143	32.7 ± 5.7	112	33.3 ± 5.3
Glucose metabolism	143		143		111	
No diabetes	87	60.4%	98	68.1%	51	35.4%
Impaired GM	38	26.4%	29	20.3%	45	31.3%
Type 2 diabetes	18	12.5%	16	11.1%	15	10.4%
Diabetes medication	144		144		144	
No	138	95.8%	135	93.8%	133	92.4%
Yes	6	4.2%	9	6.3%	11	7.6%
Lean body mass (%)	126	63.9 ± 6.3	134	67.8 ± 7.4	134	68.5 ± 7.9
Total abdominal body fat (mm²)	143	54,592.7 ± 13,414.5	143	43,838.1 ± 12,817.6	107	44,916.1 ± 11,391.4
HbA1c (%)	143	5.8 ± 0.8	143	5.9 ± 0.8	106	5.6 ± 0.6
ISI	140	0.06 ± 0.03	140	0.08 ± 0.04		
HOMA	142	2.9 ± 2.7	143	1.6 ± 1.0	109	2.1 ± 1.5
Disposition index	138	0.6 ± 0.3	141	0.4 ± 0.2	110	0.5 ± 0.3
Insulin secretion (A.U.)	138	0.57 ± 0.30	141	0.38 ± 0.20	110	0.44 ± 0.23
Fatty liver index	141	81.0 ± 18.6	143	54.7 ± 28.7	101	61.4 ± 26.3
Live fat score	141	0.3 ± 2.2	143	−1.1 ± 1.4	107	−0.7 ± 1.7
5-AVAB (log)	143	7.5 ± 0.15	143	7.4 ± 0.10		

± Depicts standard deviation.

### Baseline analyses

#### Valerobetaine levels

The intervention had a significant effect on 5-AVAB, with log-transformed 5-AVAB peak intensity decreasing slightly from T-3 to T0 (5% change, *p* = 0.019, two-sided paired *t*-test).

#### Metabolic parameters

Prior to the standardized weight reduction (T-3), 5-AVAB levels showed no significant association with any metabolic parameters assessed. At T0, however, 5-AVAB levels were moderately associated with BMI (*r* = 0.192, *p* = 0.030) and whole body fat mass (*r* = 0.192, *p* = 0.030) while negatively correlated with lean body mass (*r* = −0.237, *p* = 0.007).

With respect to glucose metabolism, we detected no association with HbA1c levels or estimates of insulin secretion (AUC Insulin/AUC Glucose, disposition index—oral glucose tolerance test) at T0. However, we found a significant association with estimates of the whole body (HOMA-IR (*r* = 0.207, *p* = 0.019)) and muscular insulin resistance, which was measured by the gold standard hyperinsulinemic-euglycemic clamp (*r* = −0.249, *p* = 0.005). This indicates a relationship between circulating 5-AVAB and an overall decreased insulin sensitivity in humans.

The liver fat score (LFS) was significantly associated with 5-AVAB levels (*r* = 0.194, *p* = 0.028) at T0. We observed a similar association with the fatty liver index (FLI) (*r* = 0.204, *p* = 0.021). For all correlations, see Table 2.

**Table 2:** Partial correlations.

Time		5-AVAB	BMI	LBM	TABF	HbA1c	ISI	HOMA	INS	DI	FLI	LFS
T-3	5-AVAB	–	0.094	−0.068	0.100	0.121	0.050	−0.060	−0.109	−0.122	0.017	0.053
T0	5-AVAB	–	0.192^a^	−0.237^b^	0.192^a^	0.044	−0.249^b^	0.207^a^	0.117	0.109	0.204^a^	0.194^a^

Control variables: Age & Sex: 5-AVAB: 5-AVAB (log-transformed).

*LBM* lean body mass, *TABF* total abdominal body fat, *ISI* insulin sensitivity index, *HOMA* homeostasis model assessment index, *INS* *insulin secretion (AU)*, *DI* disposition index, *FLI* fatty liver index, *LFS* liver fat score.

^a^Correlation is significant at the 0.05 level.

^b^Correlation is significant at the 0.01 level.

### Predictive analyses

Of the 143 participants included in the analysis, 104 completed the RCT to the primary endpoint at T18 (46 individuals in the control group and 58 in the intervention group). In correspondence to the original results, BMI was not different between both groups after 18 months (33.1 ± 5.2 kg/m^2^ vs. 33.6 ± 5.5 kg/m^2^, *p* = 0.55).

To analyze the predictive impact of 5-AVAB at T0 on BMI, HbA1c, and LFS 18 months after weight loss, we performed a linear regression analysis adjusting for age, sex, and randomization group. For a second analysis, we additionally adjusted for the baseline level of the respective covariate of interest as well as BMI change from T0 to T18 (BMI_T0T18_, for HbA1c and LFS).

5-AVAB significantly predicted future HbA1c levels (beta 0.280, *p* = 0.001). Model specifications with additional corrections and standardized beta coefficients are summarized in Table 3. This effect also persists after additional adjustment for baseline HbA1c and change in BMI_T0T18_. A dot plot of individual 5-AVAB and HbA1c levels is supplied in Supplemental Fig. 3.

**Table 3:** Regression analysis: predictors of HbA1c.

Outcome variable	Model		Correlation	St. Beta	Correlation × St. Beta × 100	*p*
HbA1c	1	(Constant)				0.103
		5-AVAB	0.318	0.280	8.897	0.001**
		Age	0.431	0.400	17.248	<0.001**
		Sex	0.081	0.067	0.549	0.414
		Randomization	−0.196	−0.165	3.231	0.048*
	Total *r*²	0.324				
	Adj. *r*²	0.297				
	2	(Constant)				0.039*
		5-AVAB	0.326	0.261	8.517	0.001**
		Age	0.371	0.312	11.547	<0.001**
		Sex	0.032	0.024	0.076	0.753
		Randomization	−0.182	−0.139	2.522	0.069
		BMI change	0.221	0.169	3.727	0.026*
		T0 HbA1c	0.377	0.318	12.003	<0.001**
	Total *r*²	0.449				
	Adj. *r*²	0.416				

St. Beta = standardized beta. Asterisks indicate statistical significance (*p < 0.05, **p < 0.01, ***p < 0.001).

To evaluate this relationship further, we performed a multinomial logistic regression analysis for 5-AVAB tertiles with respect to the impaired glucose metabolism based on oral glucose load after 18 months (no diabetes vs. impaired glucose metabolism/impaired fasting glucose and diabetes).

This analysis showed an odds ratio of 1.988 (*p* = 0.019) for impaired glucose metabolism and 3.26 (*p* = 0.027) for diabetes with increasing 5-AVAB levels. For model specifications, see Table 4.

**Table 4:** Nominal regression analyses: predictors of impaired glucose metabolism.

		*p*	OR	95% CI	
				LL	UL
Diabetes	Intercept	0.003			
	5-AVAB	0.027	3.260	1.144	9.294
	Age	0.434	1.034	0.951	1.125
	Sex	0.373	0.435	0.070	2.710
	Randomization	0.082	0.233	0.045	1.203
	BMI change	0.098	1.366	0.944	1.979
	T0 glucose metabolism	0.000	40.673	7.685	215.253
Impaired glucose metabolism	Intercept	0.022			
	5-AVAB	0.019	1.998	1.119	3.567
	Age	0.806	1.005	0.966	1.046
	Sex	0.828	0.885	0.294	2.668
	Randomization	0.274	0.589	0.228	1.521
	BMI change	0.255	1.112	0.926	1.334
	T0 glucose metabolism	0.002	7.942	2.210	28.545

**Fig. 1:**
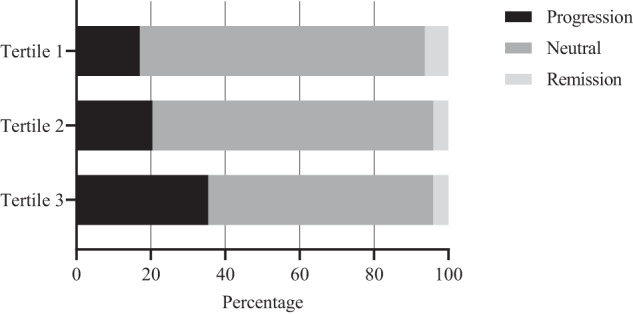
Change in diabetes status: 5-AVAB tertiles. 5-AVAB tertiles calculated from log-transformed valerobetaine values. Diabetes status is classified as 1 = “no diabetes”, 2 = “impaired fasting glucose or impaired glucose tolerance” or 3 = “type 2 diabetes mellitus”. “Progression” is defined as an upward change in status from T0 to T18, and “Remission” is defined as a downward change.

**Fig. 2:**
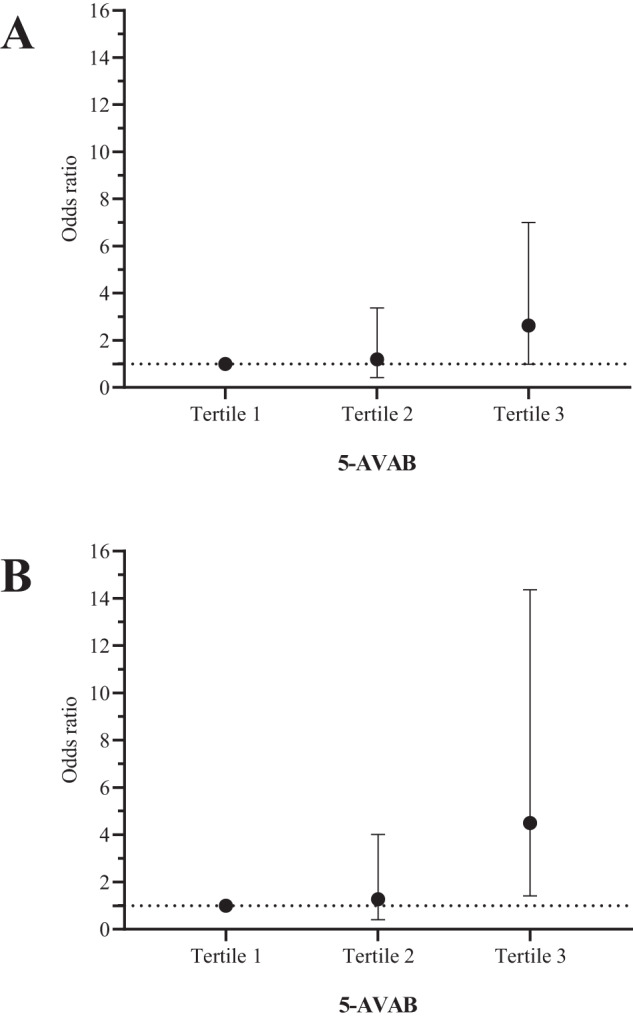
Risk of diabetes according to circulating 5-AVAB levels. Results of multinominal logistic regression analyses. **A** Crude model adjusted for age, sex, and randomization, **B** Fully adjusted model additionally considering BMI change and T0 diabetes status. 5-AVAB tertiles calculated from log-transformed 5-AVAB values.

When looking at the relative progression or remission between normoglycemia, impaired glucose metabolism, and diabetes, we found a progression rate of around 17.0% for the first tertile, while in the third tertile, this percentage doubled to 35.4% (Fig. 1). The corresponding odds ratios and model specifications are presented in Fig. 2 and summarized in Table 5.

**Table 5:** Nominal regression analysis: predictors of impaired glucose metabolism (progression).

	*p*	OR	95% CI	
			LL	UL
5AVAB Tertile^a^	0.023			
5-AVAB Tertile 2	0.676	1.277	0.407	4.010
5-AVAB Tertile 3	0.011	4.497	1.407	14.366
Age	0.741	1.007	0.967	1.048
Sex	0.501	0.694	0.239	2.013
Randomization	0.072	0.437	0.178	1.075
BMI change	0.152	1.142	0.952	1.369
T0 glucose metabolism	0.003	0.196	0.068	0.566
Constant	0.247	4.980		

^a^Reference: Tertile 1.

We repeated our analysis after the exclusion of patients medically treated for diabetes in order to avoid the confounding effect of the medication on HbA1c levels and to exclude variation due to the potential effect of a metformin-associated increase in 5-AVAB levels [15]. The reported relationship between 5-AVAB levels and HbA1c as well as impaired glucose metabolism persisted in this subset of patients. Additionally, 5-AVAB levels at T0 now showed a significant predictive value for T18 LFS (beta 0.303, *p* = 0.002). Before the correction, 5-AVAB presented only a trend towards a predictive value (beta 0.179, *p* = 0.086), which did not reach significance.

## Discussion

In summary, our data show that AVAB serum levels correlate with estimates of obesity, glucose metabolism, and hepatic steatosis, but only after the initial weight loss period. Another key finding is that 5-AVAB levels after weight loss are an independent predictor of impairments in glucose metabolism 18 months after successful weight reduction.

In the present study, a standardized weight loss intervention revealed associations of 5-AVAB to visceral obesity, insulin resistance, and hepatic steatosis. While the association with markers of adiposity and NAFLD has been described by Liu et al. [8], our data additionally show associations of 5-AVAB with markers of glucose tolerance, including ISI as measured by the gold standard hyperinsulinemic-euglycemic clamp. Insulin secretion was not associated with 5-AVAB. This supports the hypothesis of the role of 5-AVAB in the formation of insulin resistance.

As ISI data was not available for T18, we focused the further analysis on HbA1c levels and impaired glucose metabolism as composite measures. While HbA1c levels were not significantly associated with 5-AVAB in the initial analysis at T-3 and T0, the association reached significance when excluding patients receiving antidiabetic drug treatment.

The linear association to HbA1c levels at the end of the study as well as the positive odds ratio for a progressively impaired glucose metabolism support our findings at baseline.

Consistent with this, the significant associations and predictions in our study became apparent only after an initial period of weight loss, which also significantly reduced 5-AVAB. This change in 5-AVAB levels observed in our study is congruent with previous studies showing changes in microbial metabolism and metabolites following weight loss [16–18] as well as studies describing the influence of several nutrients on 5-AVAB levels [5–7]. It has to be noted that while the participants were assigned to a strict diet and showed appropriate weight loss and metabolic changes [9, 14, 19], there are no dietary assessment reports available, limiting our insight into the real-world diet of the patients.

Even with a controlled diet, this variety of dietary influences on 5-AVAB may make it difficult to study its role in human metabolism. 5-AVAB concentrations are influenced by foods that otherwise vary widely in their overall metabolic effects. Dairy products and meat from ruminants contain significant amounts of 5-AVAB [5]. However, 5-AVAB levels also increase after consumption of whole-grain products [7], which themselves do not contain 5-AVAB [20].

It should be noted that our findings regarding glucose metabolism are markedly different from studies investigating betainized compounds after dietary whole-grain interventions [7], especially considering that whole-grain intake is associated with a lower risk of T2DM [21]. As various microbial species produce 5-AVAB and alter its concentration in the circulation, these may be two different mechanisms of action [8]. In general, there are various conflicting reports regarding the effects of 5-AVAB, including proposed positive effects on fetal brain development and cancer risk, contrasting a possible detrimental role in adult metabolism [4]. Still, most of these findings are still controversially discussed. While many betainized components seem to have a positive effect on fetal neuronal growth [22], 5-AVAB seems to be associated with the pregnancy complication of pre-eclampsia [23, 24]. This highlights the need for further investigation into different mechanisms of action behind these findings. While our data are not suitable for delineating individual sources and components of the measured 5-AVAB concentrations, the preceding weight loss intervention may still provide an approach to standardize the nutritional and metabolic framework to some extent.

Generally, as our data is not quantitative and based on untargeted metabolomics, targeted prospective studies are necessary to further explore the effects of 5-AVAB on the development of metabolic diseases.

While this study is limited by the number of participants, the lack of data on the microbiome, and the associative nature of our findings, our data benefit from deep phenotyping of participants, standardized procedures including a hyperinsulinemic-euglycemic clamp, and prospective analysis.

Our results are consistent with recent experimental findings and support the relevant role of 5-AVAB in the development of metabolic syndrome while emphasizing the dietary effects on its use as a potential clinical marker. Future use of 5-AVAB measures in the clinical management of T2DM and metabolic syndrome will most likely require an integrated interpretation in the context of diet and microbiome.

### Supplementary information

Supplementary Figure Legends

Supplementary Figure 1

Supplementary Figure 2

Supplementary Figure 3

Supplementary Text 1

## Data Availability

The data that support the findings of this study are available from the authors, but restrictions apply specifically to the retention time database of metaSysX. This information is part of their most important intellectual property, therefore they cannot provide the raw files or any other files that contain retention time information. Other data are however available from the authors upon reasonable request and with permission of metaSysX.
