# Supplementary material for: 5-Aminovaleric acid betaine predicts impaired glucose metabolism and diabetes
Source: Nutr Diabetes. 2023 Sep 20;13:17. doi: 10.1038/s41387-023-00245-3 (PMC10511423; doi:10.1038/s41387-023-00245-3)
Supplement: Supplementary file 1 — Supplementary Figure Legends [file 41387_2023_245_MOESM1_ESM.docx]

**Supplemental Figure 1**

*Timescale of the randomized controlled trial*

Visualization of the Maintain-Adults trial timeline. Created with Biorender.com.

**Supplemental Figure 2**

*Fragmentation spectrum and extracted ion chromatogram*

The peak of VB eluted at 0.95 min and the fragmentation spectrum of m/z 160.1332 acquired upon HCD in the collision energy 25 V. The fragmentation pattern for the samples corresponds to the fragmentation spectrum acquired for a true standard.

**Supplemental Figure 3**

*Individual 5-AVAB and HbA1c levels*

Dot plot of individual 5-AVAB levels at baseline (T0) and HbA1c (%) levels 18 months later (T18). The dashed line represent the regression curve.
