# Supplementary figures and images for: 5-Aminovaleric acid betaine predicts impaired glucose metabolism and diabetes
Source: Nutr Diabetes. 2023 Sep 20;13:17. doi: 10.1038/s41387-023-00245-3 (PMC10511423; doi:10.1038/s41387-023-00245-3)

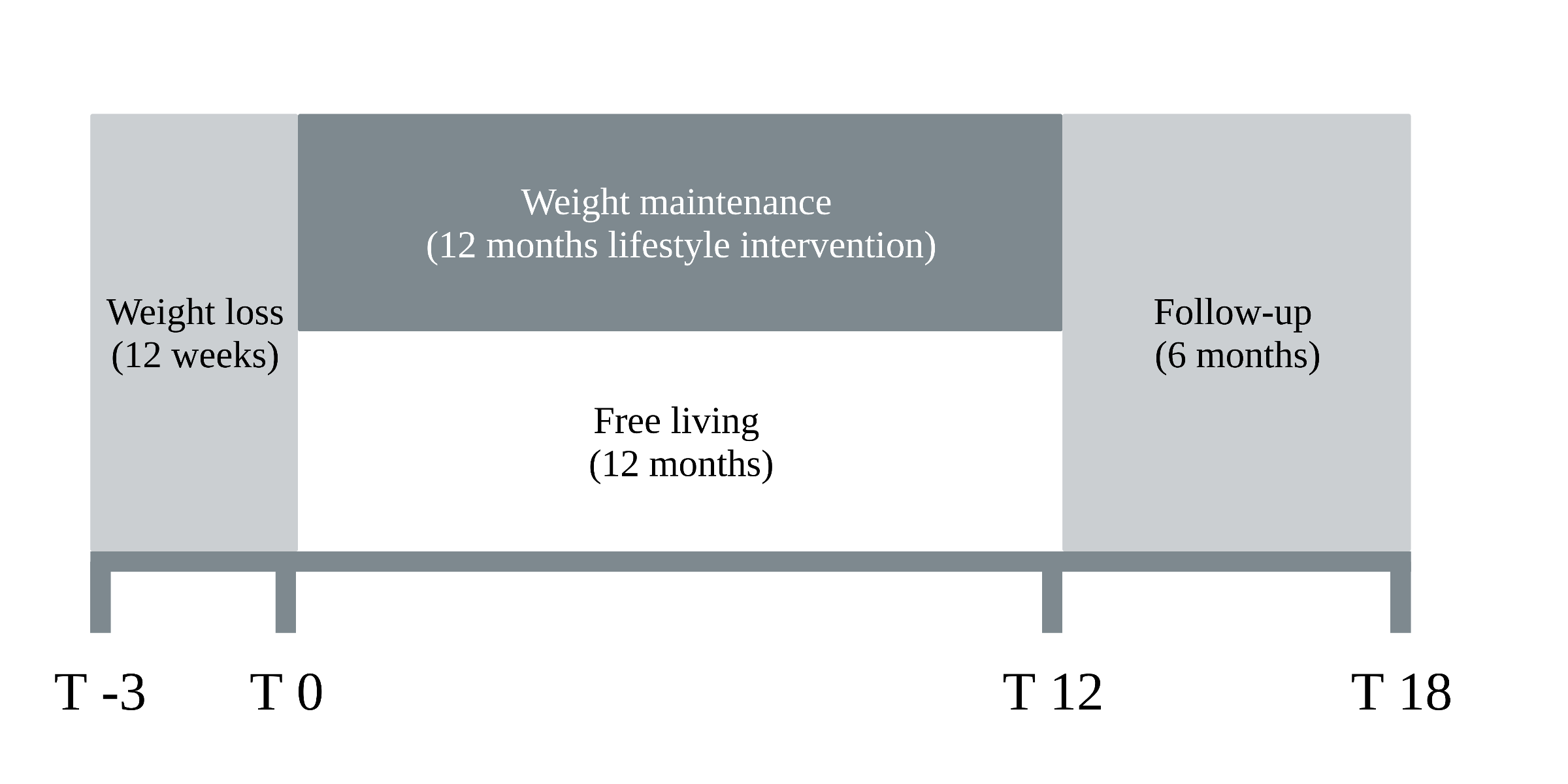

Supplement: Supplementary file 2 — Supplementary Figure 1 [file 41387_2023_245_MOESM2_ESM.png]
