# Supplementary material for: 5-Aminovaleric acid betaine predicts impaired glucose metabolism and diabetes
Source: Nutr Diabetes. 2023 Sep 20;13:17. doi: 10.1038/s41387-023-00245-3 (PMC10511423; doi:10.1038/s41387-023-00245-3)
Supplement: Supplementary file 5 — Supplementary Text 1 [file 41387_2023_245_MOESM5_ESM.pdf]

Supplementary Material

Methods - Untargeted Metabolomics

The mass spectrometry-based metabolome analysis was performed by the company metaSysX GmbH, Potsdam, Germany.

LC-MS sample preparation and extraction

The sample preparation was performed according to metaSysX procedure, a modified protocol from Salem *et al.*[1]. Briefly, 50 µl of material was used for extraction of polar and lipophilic compounds by methyl-tert-butyl-ether (MTBE)/methanol/water. The organic phase containing lipids and lipophilic compounds was transferred into the 1.5 ml tube. 500 µl and 150 µl of aqueous phase containing semi-polar and polar compounds was transferred into the new tubes for LC- and GC-MS analysis, respectively. The collected phases were dried down in vacuum concentrator and stored in -80 °C until analysis.

LC-MS

The dried samples were dissolved in 100 µl of water or acetonitrile for polar and lipid measurements respectively. 2 µl were injected for the LC-MS analysis. The analysis was performed using QExactive Orbitrap MS (Thermo Fisher Scientific) coupled to the ultraperformance liquid chromatography (UPLC).

Liquid phase chromatography

The samples were measured with a Waters ACQUITY Reversed Phase Ultra Performance Liquid Chromatography (RP-UPLC) coupled to a Thermo-Fisher QExactive mass spectrometer. BEH C_8 (ACQUITY UPLC BEH Column, 130Å, 1.7 µm, 2.1 mm X 100 mm) and HSS T3 C_{18} (ACQUITY UPLC HSS T3 Column, 100Å, 1.8 µm, 2.1 mm X 100 mm) columns were used for the lipophilic and the hydrophilic measurements, respectively. A 15 min gradient was used for separation of polar and lipophilic compounds. The mobile phases for separation of polar and semi-polar compounds were 0.1% formic acid in H_2O (buffer A) and 0.1% formic acid in acetonitrile (buffer B). Following conditions

were used for chromatographic separation: A 95% to A 60% to 11 min, A 60% to A 30% from 11 to 13 min and A30% to A 1% from 13 to 15 min. For the separation of lipids and lipophilic metabolites following mobile phases were used: 1% of 1M NH_4Ac in 0.1% acetic acid (buffer A) and acetonitrile: isopropanol (7:3) containing 1% of 1M NH_4Ac in 0.1% acetic acid (buffer B) with the step gradient of 45% to 25 % A from 1 to 4 min, 25% to 11% A from 4 to 12 min and 11% to 0% A from 12 to 15 min.

Mass spectrometry

All mass spectra were acquired in Full Scan MS (Mass Range [100-1500]) positive and negative ionization mode with the following settings of the instrument: Heated electrospray ionization (HESI) was used, spray voltage was 3.5 kV, capillary temperature 275 °C, sheath gas flow rate 60 units, mass resolving power 70000, 3e6 target value (AGC) and maximal fill time of 200 ms.. To increase number of identified compounds pooled samples were measured in LC-MS/MS mode. The full mass spectrum was acquired for mass range 100-1500 every three MS/MS scans that were recorder for mass range 100-1500. The higher-energy collisional dissociation (HCD) fragmentation spectrum was recorded for three most intense precursor ions at normalized collision energy 25. The mass resolution was set to 35000 and 17500, the AGC to 1e5 and 5e4 ions, and maximal fill time of 100 ms and 50 ms for MS and MS/MS, respectively. The dynamic exclusion was set to 3s. Thermo Excalibur was used for the data acquisition.

LC-MS data annotation

metaSysX database of chemical compounds was used to annotate the features detected in the LC-MS polar and non-polar platform. The metaSysX database contains the mass-to-charge ratio and the retention time information of reference compounds measured at the same chromatographic and spectrometric condition as samples measurements. 7 ppm and 0.1 min deviation from the reference compounds mass-to-charge-ratio and retention time were allowed as matching criteria for polar and lipid platform. Coeluting compounds with the same mass of generated ion were kept as conflicting annotation. Annotated lipids were confirmed using R-based algorithm developed in metaSysX which uses MS/MS fragmentation spectrum.

Reference list

[1] Salem MA, Jüppner J, Bajdzienko K, Giavalisco P. Protocol: a fast, comprehensive and reproducible one-step extraction method for the rapid preparation of polar and semi-polar metabolites, lipids, proteins, starch and cell wall polymers from a single sample Plant Methods. 2016 Nov 10; DOI: 10.1186/s13007-016-0146-2
